# Call for participation in a multicenter study: Intraoperative blood loss – Towards a standardized and objective recording method

**DOI:** 10.1016/j.sopen.2025.07.007

**Published:** 2025-08-05

**Authors:** Emelie Nébel, Lätitia Dennin, Johannes Klose, Ulrich Ronellenfitsch, Jörg Kleeff, Artur Rebelo, the Young Surgeons Publications Committee, Patrick S. Plum, Martina Nebbia, Thomas F. Stoop, Mara R. Goetz, Lena Conradi, Nathanael Raschzok, Hannes Jansson, Patrik Larsson, Sarah Gerber, Jorge Roldàn, Theodoros Michelakos, Benedict Kinny-Köster, Ingmar F. Rompen, Oskar Franklin, Ioannis A. Ziogas, Hiroyuki Ishida, Andrii Khomiak, Daisuke Hashimoto, Vincent P. Groot, Y.H. Andrew Wu, Toshitaka Sugawara, Atsushi Oba, Aya Maekawa, Jelmer E. Oor, Alessio Morandi, David B. Stewart, Nicholas Joseph Zyromski, Matthias Ilmer, Ibrahim Büdeyri, Artur Rebelo, Constantin Scholz, Elisa Bannone, Krishna Kotecha, Adisa Poljo

**Affiliations:** 1 Department of Visceral, Transplant, Thoracic and Vascular Surgery, University Hospital Leipzig, Leipzig, Germany; 2 Division of Surgery and Oncology, Department of Clinical Science, Intervention and Technology, Karolinska Institutet, Stockholm, Sweden; 3 Amsterdam UMC, location University of Amsterdam, Department of Surgery, Amsterdam, the Netherlands; 4 Cancer Center Amsterdam, Amsterdam, the Netherlands; 5 Department of General, Visceral and Thoracic Surgery, University Medical Center Hamburg-Eppendorf, Hamburg, Germany; 6 Department of Surgery, University Medical Center Goettingen, Goettingen, Germany; 7 Department of Surgery, Campus Charité Mitte | Campus Virchow-Klinikum, Charité– Universitätsmedizin Berlin, Freie Universität Berlin, Humboldt-Universität zu Berlin, BIH Biomedical Innovation Academy, BIH Charité Clinician Scientist Program, Berlin Institute Health at Charité– Universitätsmedizin BerlinCharité, Germany; 8 Department of Surgery, Inselspital Bern, Bern, Switzerland; 9 Department of HPB Surgery, Hospital Angeles Universidad Mexico City, Mexico City, Mexico; 10 Department of Surgery, University of Chicago, US; 11 Department for General, Visceral and Transplantation Surgery, Heidelberg University Hospital, Heidelberg, Germany; 12 Department of Diagnostics and Intervention, Surgery, Umeå University, Umeå, Sweden; 13 University of Colorado Anschutz Medical Campus, Aurora, USA; 14 Department of Pancreatobiliary Surgery, Kansai Medical University, Osaka, Japan; 15 Department of Surgery, Regional Academic Cancer Center Utrecht, UMC Utrecht Cancer Center, St Antonius Hospital Nieuwegein, Utrecht, the Netherlands; 16 Division of Vascular Surgery and Endovascular Therapy, Department of Surgery, The Johns Hopkins School of Medicine, Baltimore, USA; 17 Department of Surgery, Albany Medical Center, Albany, USA; 18 Department of Hepatobiliary and Pancreatic Surgery, The Institute Science Tokyo, Tokyo, Japan; 19 Division of Hepatobiliary and Pancreatic Surgery, Cancer Institute Hospital, Japanese Foundation for Cancer Research, Tokyo, Japan; 20 Department of Surgery, Oncology and Gastroenterology (DiSCOG), Padova University Hospital, Padova, Italy; 21 Department of Surgical Oncology, University Medical Center Utrecht, the Netherlands; 22 Hepato-Pancreato-Biliary (HPB) Unit, Careggi Hospital, Florence, Italy; 23 Department of Surgery, Simmons Center Institute, Springfield, USA; 24 Department of Surgery, Indiana University school of Medicine, Indianapolis, USA; 25 Division of General, Abdominal, and Transplant Surgery, Ludwig-Maximilian University Munich, Munich, Germany; 26 Department of General, Visceral and Transplant Surgery, University Hospital Muenster, Muenster, Germany; 27 Department of Surgery, University Hospital Halle, Halle, Germany; 28 Department of Surgery, University Medicine Mainz, Mainz, Germany; 29 Department of Hepato-Bilio-Pancreatic Surgery- P. Pederzoli Hospital, Peschiera Del Garda, Verona, Italy; 30 Department of Surgery, Royal North Shore Hospital, University of Sydney, Australia; 31 University Digestive Health Care Center Basel, Clarunis, Switzerland; Department of Visceral, Vascular and Endocrine Surgery, Martin-Luther-University Halle-Wittenberg, University Medical Center Halle (Saale), Ernst-Grube-Str. 40, 06120 Halle (Saale), Germany

## 1 Background

Intraoperative blood loss is one of the key challenges in surgical practice and remains a critical parameter in almost every surgical procedure. Despite its obvious importance for perioperative management, a generally accepted standardized method for precise estimation of blood loss in the operating room is not available. Currently paracticed approaches are often inaccurate, subjective and vary considerably between hospitals [1]. Inaccurate recording of blood loss may bear serious consequences for treatment decisions and outcome as well as morbidity and mortality of surgical patients [2]. Despite a strong clinical rationale, there is still no standardized, evidence-based method to reliably measure actual blood loss during surgery. In daily practice, estimates are often based on subjective assessments of visual findings such as blood in the surgical field, in suction cups or on drapes. However, visual estimation is regularly inaccurate and does not become more accurate with increasing professional experience [3]. Smaller amounts of blood are often overestimated and larger amounts tend to be underestimated [4]. Nevertheless, visual estimation is common practice due to its low cost and effort [1]. Another method of estimating blood loss is to weigh used dressing material before and after use in the operating room [5]. According to a study from 2020, this method is only used in a few hospitals (8 %), despite its ease of use [1]. Another method to determine intraoperative blood loss is to calculate it using mathematical formula that require the circulating blood volume per patient, the pre- and postoperative haematocrit value and haemoglobin value.

Inaccuracies in estimating intraoperative blood loss may not only have an impact on direct patient care, but also make scientific analyses, quality management and the development of uniform guidelines for blood loss and transfusion management as well as perioperative therapy more difficult. Particularly in an era of increasing patient safety, resource optimization and evidence-based medicine, we believe it is essential to address this deficit.

In studies addressing intraoperative blood loss in patients undergoing pancreatic or liver resection from 2006 to 2021, 83 % of the studies did not even specify how blood loss was measured. Yet 83 % of respondents wanted standardization in HPB surgery. [6] Therefore, standardization of intraoperative blood loss assessment is urgently needed.

## 2 Planned approach

In our current study, we are systematically investigating various approaches of measuring intraoperative blood loss. The aim of this first survey is to shed light on the existing problem, to emphasize its clinical relevance and to develop eay-to-implement approaches for reliable quantification.

Patients at the Department of Visceral, Vascular and Endocrine Surgery, University Hospital Halle (Saale), are being prospectively enrolled in the study. Included are patients older than 18 years who undergo surgery in one of the following departments: HPB, colorectal, general/visceral surgery, vascular surgery and thoracic surgery. Furthermore, the expected intraoperative blood loss should be higher than 100 mL. Patients under 18 years of age, with continuous dialysis, with extracorporeal life support and patients undergoing cardiac surgery are excluded from the study. We will collect the subjective visually quantified blood loss based on surgeons and anaesthetists' perception of the surgical field after the surgery. Furthermore, the measured amount of blood loss is documented by weighing the used swabs and sponges before and after use and the amount of aspirated secretion during the operation. The intraoperative blood loss is also calculated with three known formulas, using the pre- and postoperative haemoglobin, haematocrit and circulating blood volume.

A retrospective analysis of patients who are older than 18 years underwent surgery at the Department of Visceral, Vascular and Endocrine Surgery, University Hospital Halle (Saale) between 2017 and 2025 is currently being conducted.

## 3 First results

To date, 71 datasets have been prospectively collected from patients. The median blood loss visually estimated by the surgeon was 350 mL (IQR 475 mL), while the estimate by the anaesthetist was slightly higher, with a median of 400 mL (IQR 625 mL). In 39 of these cases, blood loss was also calculated based on the weight of sponges, swabs, and abdominal drapes, combined with the volume in the suction container. This method yielded a median blood loss of 555.57 mL (IQR 767.1 mL). For the same 39 datasets, the median blood loss calculated using the formula proposed by Good et al. was 1070.97 mL (IQR 646.5 mL).

**Fig. 1:**
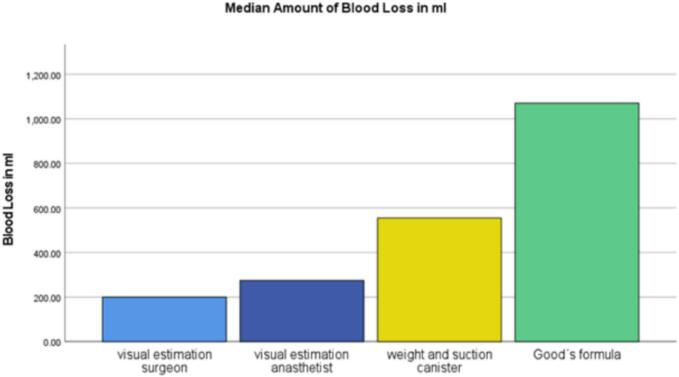
Median amount of blood in mL (*n* = 39).

**Fig. 2:**
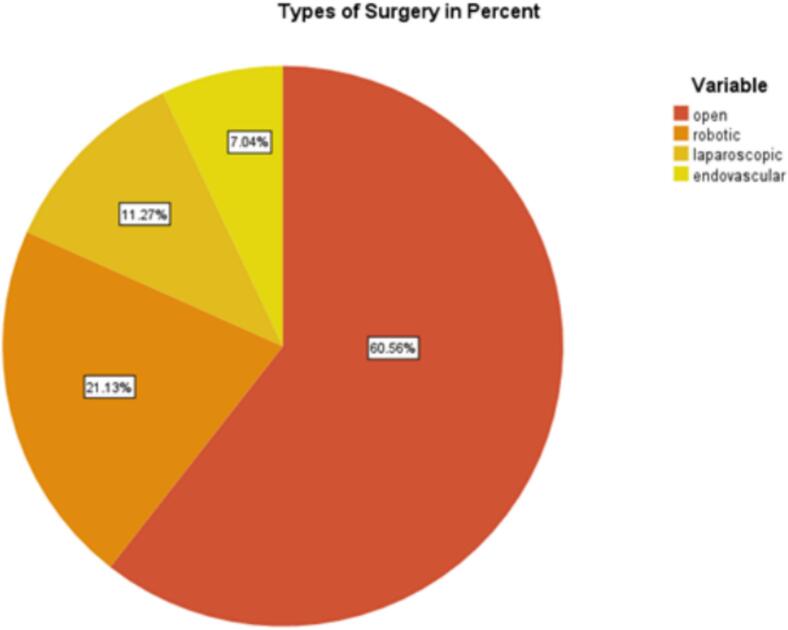
Types of surgery in percent (*n* = 71).

The first results show that anaesthesiologists visually estimate a higher blood loss as compared to surgeons, but both estimates are below the values of the weighed volume and the calculated volume according to Good et al. Furthermore, surgeons more frequently underestimated blood loss as compared to anaesthesiologists. Conversely, anaesthesiologists overestimate blood loss more often than surgeons. The inaccuracy and interpersonal variability of visual estimation mentioned in many studies can be confirmed (Fig. 1, Fig. 2).

Further subgroup analyses as well as the evaluation of feasibility and implementation costs will be conducted in the retrospective cohort.

## 4 Call for participation in a multicenter study

We would like to cordially invite you to actively participate in the next step: a comprehensive survey to design a prospective multicenter trial!

A collaboration network of multiple centers will allow us to create a broad, reliable database that is suitable for developing evidence-based recommendations in order to set new standards.

The first step will be a multicenter survey to compare different measurement methods and surgical settings, further allowing us to raise awareness for this topic in our surgical community.

The basic work of our current study is intended to serve as a pilot study for the larger multi-center project. We are therefore looking for centers to participate in a comprehensive survey. The survey will allow us to design a robust multicenter prospective trial for assessing intraoperative blood loss estimates. This study aims to set standards for a homogeneous future blood loss estimation algorithm.

If you are interested in participating or would like to receive further information regarding the planned study, please do not hesitate to contact us.

We are looking forward to creating a strong surgical network to set surgical standards and advance in our field!

## Author contribution

All authors critically revised the manuscript and approved the final version.

## Credit authorship contribution statement

**Emelie Nébel:** Writing – original draft. **Lätitia Dennin:** Writing – review & editing. **Johannes Klose:** Writing – review & editing. **Ulrich Ronellenfitsch:** Writing – review & editing. **Jörg Kleeff:** Writing – review & editing. **Artur Rebelo:** Conceptualization, Methodology, Writing – review & editing.

## Ethics approval

The study was approved by the Ethics Committee of Martin-Luther-University Halle-Wittenberg under reference number: 2023–156. Compliance with ethical standards.

## Funding sources

No external funding was received for this study.

## Declaration of competing interest

The authors declare that there is no conflict of interest.
